# Determinants of blood pressure control amongst hypertensive patients in Northwest Ethiopia

**DOI:** 10.1371/journal.pone.0196535

**Published:** 2018-05-02

**Authors:** Destaw Fetene Teshome, Amsalu Feleke Demssie, Berihun Megabiaw Zeleke

**Affiliations:** 1 Department of Epidemiology and Biostatistics, Institute of Public Health, University of Gondar, Gondar, Ethiopia; 2 Department of Health Service Management and Health Economics, Institute of Public Health, University of Gondar, Gondar, Ethiopia; 3 Department of Epidemiology and Preventative Medicine, School of Public Health and Preventative Medicine, Monash University, Melbourne, Victoria, Australia; The University of Tokyo, JAPAN

## Abstract

**Background:**

Controlling blood pressure (BP) leads to significant reduction in cardiovascular risks and associated deaths. In Ethiopia, data is scarce about the level and determinants of optimal BP control among hypertensive patients. This study aimed to assess the prevalence and associated factors of optimal BP control among hypertensive patients attending at a district hospital.

**Methods:**

A hospital-based, cross-sectional study was conducted among 392 hypertensive patients who were on treatment and follow-up at a district hospital. A structured questionnaire adopted from WHO approach was prepared to collect the data. Medication adherence was measured by the four-item Morisky Green Levine Scale, with a score ≥3 defined as “good adherence”. Blood pressure was measured, and optimal BP control was 0DEFined as systolic BP < 140 mmHg and diastolic BP<90 mmHg. Both binary and multivariable logistic regressions models were fitted to identify correlates of optimal BP control. All statistical tests were two-sided and a p values <0.05 was considered for statistical significance.

**Results:**

The mean age of the participants was 58 years (SD±13 years). Over half (53.8%) were females. Three quarters (77.3%) of the participants were adherent to their medications. The overall proportion of participants with optimally controlled BP was 42.9%.Female sex (Adjusted Odd Ratio(AOR) = 1.94, 95% CI: 1.15, 3.26), age older than 60 years (AOR = 2.95, 95% CI: 1.18, 7.40), consumption of vegetables on most days of the week (AOR = 2.16, 95% CI: 1.25, 3.73), physical activity (AOR = 4.85, 95% CI: 2.39, 9.83), and taking less than three drugs per day (AOR = 3.04, 95% CI: 1.51, 6.14) were positively associated with optimally controlled BP. Poor adherence to medications (AOR = 0.18, 95% CI: 0.09, 0.35), having asthma comorbidity (AOR = 0.33, 95% CI:0.12, 0.88) and use of top added salt on a plate (AOR = 0.20, 95% CI:0.11, 0.36) were negatively associated with optimal BP control.

**Conclusion:**

A higher proportion of hypertensive patients remain with un-controlled BP. Modifiable risk factors including poor adherence to medications, lack of physical exercise, adding salt into meals, being on multiple medications and comorbidities were significantly and independently associated with poor BP control. Evidence-based, adherence-enhancing and healthy life style interventions should be implemented.

## Introduction

High blood pressure or hypertension, being an established modifiable risk factor for cardiovascular disease (CVD) morbidity and mortality [1] is a global public health issue [2, 3]. Globally, hypertension is the second most important preventable risk factor contributing to 13% of deaths [4–6]. The prevalence of hypertension has ever been increasing at an alarming rate among developing nations, especially the African region. In Ethiopia, 46% of adults aged 25 years or older are reported to have high BP [4] making hypertension the 7^th^leading cause of death (3.5% of all deaths) in the country [7].

Long-term BP lowering to what is considered either normal or optimal levels results in multiple health benefits. For instance, effective and sustained lowering of the BP of hypertensive patients by 2 mmHg reduces the risk of CVD events by up to 10% [8]. Similarly, if systolic blood pressure is managed to be lowered by 20 mmHg the risk of dying from a stroke and coronary heart disease will decrease by 50% [3].

Antihypertensive medication is one of the measures to manage hypertension. However, studies had shown that merely less than half of hypertensive patients on medications have optimal BP control [5–6, 9–14]. In sub-Saharan African urban population less than half of those being treated for hypertension had their BP levels controlled [15, 16]. Whereas a number of studies had reported the prevalence and correlates of hypertension in Ethiopia [17–20], evidence is lacking about the prevalence and determinants of BP control among hypertensive patients on treatment and follow-up at a district hospital. This study aimed to quantify the proportion of patients who had adequately controlled their blood pressure, and to identify factors associated with it.

## Methods

### Study design and participants

This hospital-based cross-sectional study was conducted at Debre Tabor district hospital, Northwest Ethiopia. The hospital is located in Debre Tabor town (667 km north-west of Addis Ababa), South Gondar Zonal administration of the Amhara National Regional State (ANRS). Currently, the hospital has a catchment population of about 2.3 million people in the zone and nearby districts.

Study participants were recruited, between March and May 2015, from the outpatient chronic illness follow up clinic of the hospital. All hypertensive patients aged 18 years or older on outpatient follow-up and anti-hypertensive medication therapy for at least 6 months were included in this study.

### Sample size determination

The sample size of 416 was determined based on a 95% confidence interval around a percentage prevalence estimate of 43.6% controlled BP [17] with a 5% margin of error and a 10% non-response rate. Since the calculated sample size was close to the target population of 421 hypertensive patients, all the 397 eligible patients were included in the study.

### Data collection tools and techniques

A structured questionnaire that included variables on sociodemographic characteristics, medical history, and other previously validated tools including the WHO STEP wise approach to surveillance non communicable diseases [21], and the Morisky Green Levine Scale were used to collect the data.

The questionnaire was pre-tested on 20 hypertensive patients from the nearby health center and modified accordingly before the main study was begun. Four data collectors and a supervisor were collected data from both primary and secondary sources (patient interviews and record reviews). Training was given for 2 days and information exchange by telephone and close supervision by the principal investigator and supervisor were made on a daily basis. Coding and data cleaning were done (checking frequencies and cross-tab for each item).

To ascertain blood pressure control, we undertook document review of patient’s charts and recorded the average of three BP levels measured over the preceding 6 months (one being the most recent).

### Operational definition

Optimally controlled BP was defined as an average systolic BP <140 and diastolic BP < 90 mmHg if the patient is younger than 60 years [8, 22] or an average systolic BP< 150 and diastolic BP < 90mmHg if patient was older than 60 years [8].

Patients with score of ≥3 (range: 0–4) on the 4-items Morisky Green Levine Scale self-reported measures of medication-taking behavior were assessed as having good adherence to medications, otherwise classified as non-adherent.

Physical activity level of the respondents was categorized into no, low, or moderate. Patients were defined as having no physical activity if they do not perform any form of physical activity for at least 10 minutes. If a patient declares performing moderate activity per week for less than 5 times of each at least 30 minutes, or less than three times of each at least 20 minutes of vigorous activity per week, then he/she was defined as having low level of physical activity. Similarly, a moderate level of physical activity was defined as if the patient performed 3 or more days of vigorous intensity activity of at least 20 minutes per day or, 5 or more days of moderate intensity activity or, walking of at least 30 minutes per day. Top added salt on plate was defined as the addition of raw salt on meal during meal preparation.

### Data processing and analysis

Data were entered using EPI INFO version 7 and analyzed by using SPSS version 20. Data were checked and cleaned for completeness and consistency of values and variables. Descriptive statistics were used to present data in text and tables. Both bivariate and multivariable logistic regressions were fitted to identify factors associated with optimal BP control. All variables with p-values up to 0.2 in the bivariate analysis were fitted in to multivariable logistic regression to control for the possible effects of confounding. Hosmer-Lemeshow goodness of test for the model was also checked. All statistical tests were two sided and variables which have significant association with the outcome variable were identified on the basis of AOR with 95% CI, p-values <0.05.

## Results

### Participant characteristics

Of the 397 eligible patients attending at the clinic in the three months of data collection, 392 participated in this study (response rate of 98.7%).

The mean age of respondents was 58 years (SD±13 years) which range from 23–88 years. Three hundred eighty seven (98.7%) of the respondents were from Amhara ethnic group, 254 (64.8%) were currently married, 211 (53.8%) were females, and 372 (94.9%) identified themselves as followers of orthodox Christianity. About half of participants (49%) were unable to read and write, and two-thirds (69.4%) were urban dwellers. The median monthly family income of the respondents was 600 Ethiopian Birr (ETB) with inter quartile range of1129 ETB (Table 1).

**Table 1:** Sociodemographic characteristics of participants (n = 392).

Variables	Frequency	Percent
Sex		
Male	181	46.2
Female	211	53.8
Age in years		
18–40	41	10.5
41–60	179	45.7
≥61	172	43.9
Ethnic group		
Amhara	387	98.7
Other	5	1.3
Religion		
Orthodox	372	94.9
Muslim	11	2.8
Others*	9	2.3
Marital status		
Single	9	2.3
Married	254	64.8
Divorced	44	11.2
Widowed	80	20.4
Separated	5	1.3
Educational status		
Unable to read and write	192	49.0
Read and write	58	14.8
Primary school	53	13.5
Secondary school	15	3.8
High school	11	2.8
College/University completed	63	16.1
Occupational status		
Housewife	133	33.9
Farmer	97	24.7
Government employed	70	17.9
Merchant	28	7.1
No job	25	6.4
Retired	22	5.6
Daily laborer	10	2.6
Other**	7	1.8
Residence		
Urban	272	69.4
Rural	120	30.6
Monthly family income***		
≤ 370	98	25.0
371–600	103	26.3
601–1500	100	25.5
>1500	91	23.2

*Adventist.

**Student, Nongovernmental organization workers.

*** Income is categorized based on quartile classification.

### Behavioral and life style characteristics of respondents

Of all participants, 384 (98%) and 112 (28.6%) were eating cereal products and vegetables on most days of the week respectively. Two hundred forty six (62.8%) of the respondents were using Sesame oil for their usual meal preparations and a third of participants (32.1%) add salt when cooking or preparing food at home.

Five patients (1.3%) were current smokers, and 48 (12.2%) reported that they drink alcohol on daily basis. Close to half of patients (44.4%) are classified as having moderate levels of physical activity.

### Co-morbidities and family history of hypertension related factors of the respondents

Of all respondents 72 (18.4%), 68 (17.3%), 30 (7.7%) and 26 (6.6%) have diabetes mellitus, cardiovascular diseases, asthma and chronic kidney diseases respectively. Eighty one (20.7%) of the respondents reported as they had family history of hypertension.

### Medication adherence and BP control

Among the respondents 285 (72.7%) of the study subjects had been taking less than 3 types of drugs per day and 303 (77.3%) were classified as having good adherence to prescribed medications (Table 2).

**Table 2:** Anti-hypertensive drugs and level of adherence for medications (n = 392).

Variables	Frequency	Percent
Types of drug/s		
HCT	115	29.3
Enalapril	45	11.5
Nifidipine	50	12.8
Atenelol	14	3.6
Methyldopa	3	0.8
HCT & Enalapril	79	20.2
HCT &Nifidipine	50	12.8
Enalapril &Nifidipine	25	6.4
HCT, Enalapril &Nifidipine	11	2.8
Number of drugs		
<3	285	72.7
≥3	107	27.3
Adherence level		
Adherent	303	77.3
Non adherent	89	22.7

The mean systolic and diastolic BP readings were 142.17 mmHg (±14.69 SD) and 87.74 mmHg (±8.69 SD). The overall proportion of optimally controlled BP was 42.9% (95% CI:38.3, 47.4%).

### Factors associated with optimal BP control

After adjustment for potential confounders (sex, age, consumption of vegetables on most days of the week, top added salt, physical activity, Cardio Vascular Diseases, Diabetes Mellitus, asthma, number of medications per day and adherence to medications),female sex, age older than 60 years, consumption of vegetables on most days of the week, adequate physical exercise, and taking less than 3 drugs per day were significantly and positively associated with optimally controlled BP whereas poor adherence to medications, use of top added salt on a plate and having asthma comorbidity were significantly and negatively associated with optimal BP control.

Females were twice (AOR = 1.94, 95% CI: 1.15, 3.26) more likely to have optimal BP control as compared with males. Compared with younger patients (aged 18–40 years), those aged over 60 years were three times (AOR = 2.95, 95% CI: 1.18, 7.40) more likely to have optimal BP control. Patients who consumed vegetables on most days of the week were two times (AOR = 2.16, 95% CI: 1.25, 3.73) more likely to have optimally controlled BP as compared to patients who didn’t consumed vegetables on most days of the week. Compared to patients with no physical activity, those who did low and adequate level of physical exercise were three times (AOR = 2.61, 95% CI: 1.28, 5.31) and five times (AOR = 4.85, 95% CI: 2.39, 9.83) more likely to have optimally controlled BP respectively. Hypertensive patients who took less than 3 drugs per day were three times (AOR = 3.04, 95% CI: 1.51, 6.14) more likely to have optimal BP control as compared to patients who took 3 or more drugs per day. Hypertensive patients who used top added salt on a plate was 80% (AOR = 0.20, 95% CI: 0.11, 0.36) less likely to have optimal BP control as compared to patients who didn’t used. Hypertensive patients who have had asthma comorbidity were 67% (AOR = 0.33, 95% CI:0.12, 0.88) less likely to have optimal BP control as compared to non-asthmatic patients.

Patients who were poorly adherent to their prescribed antihypertensive drugs were 82% (AOR = 0.18, 95%CI = 0.09, 0.35) less likely to have optimal BP control as compared to those who were adherent to their antihypertensive drugs (Table 3).

**Table 3:** Bivariate and multivariable logistic regression analysis of factors associated with optimal blood pressure control of hypertensive patients in Debre Tabor Hospital, ANRS, Northwest Ethiopia, May 2015 (n = 392).

Variables	Blood pressure control		Crude OR (95% CI)	Adjusted OR (95% CI)
	Controlled	Uncontrolled		
Sex				
Male	71 (39.2%)	110 (60.8%)	1	1
Female	97 (46.0%)	114 (54.0%)	1.32 (0.88, 1.97)	1.94 (1.15, 3.26)*
Age				
18–40	14 (34.1%)	27 (65.9%)	1	1
41–60	70 (39.1%)	109 (60.9%)	1.24 (0.61, 2.52)	1.41 (0.57, 3.47)
≥61	84 (48.8%)	88 (51.2%)	1.84 (0.90, 3.75)	2.95 (1.18, 7.40)*
Vegetable consumption				
Yes	66 (58.9%)	46 (41.1%)	2.50 (1.60, 3.92)*	2.16 (1.25, 3.73)*
No	102 (36.4%)	178 (63.6%)	1	1
Top added salt				
Yes	22 (17.5)	104 (82.5%)	0.17 (0.10,0.29)*	0.20 (0.11, 0.36)**
No	146 (54.9%)	120 (45.1%)	1	1
Physical activity (PA)				
No physical exercise	21 (25.0%)	63 (75.0%)	1	1
Low level of PA	55 (41.0%)	79 (59.0%)	2.09 (1.14, 3.81)*	2.61 (1.28, 5.31)*
Adequate PA	92 (52.9)	82 (47.1%)	3.37 (1.89, 5.99)*	4.85 (2.39, 9.83)**
Cardiovascular diseases				
Yes	22 (32.4%)	46 (67.6%)	0.58 (0.34, 1.01)	0.86 (0.42, 1.78)
No	146 (45.0%)	178 (55.0%)	1	1
Diabetes mellitus				
Yes	16 (22.2%)	56 (77.8)	0.32 (0.17, 0.57)*	0.57 (0.26, 1.24)
No	152 (47.5%)	168 (52.5%)	1	1
Asthma				
Yes	7 (23.3%)	23 (76.7)	0.38 (0.16, 0.91)*	0.33 (0.12, 0.88)*
No	161 (44.5%)	201 (55.5%)	1	1
Number of drug taken per day				
<3	147 (51.6%)	138 (48.4%)	4.36 (2.57, 7.42)*	3.04 (1.51, 6.14)*
≥3	21 (19.6%)	86 (80.4%)	1	1
Adherence to medication				
Adherent	150 (49.5%)	153 (50.5%)	1	1
Poor adherent	18 (20.2%)	71 (79.8%)	0.26 (0.15, 0.46)*	0.18 0.09, 0.35)***

*P-value<0.05.

**P-value<0.001.

Hosmer and Lemeshow Test = 0.664.

## Discussion

Controlling blood pressure in people with hypertension to reduce cardiovascular morbidity and mortality is a major challenge public health problem in many developing countries including Ethiopia.

This study revealed that only 42.9% of hypertensive patients had optimally controlled BP despite being on follow up at a hospital. This finding was in line with studies done in Adama Hospital Medical College Ethiopia (43.6%) [16], urban-rural China (45.9%) [11], and rural India (46.9%) [5]. It was higher than studies done in rural and urban communities in high, middle, and low income countries (32.5%) [9], Kerala-India (25%) [10], Vietnam (36.3%) [13] and Bangladesh (31.4%) [14]. However, it was lower than the studies done in Asian-Indians (48.7%) [9], Macau-China (49%) [12], and Sudanese adults (64%) [23]. These difference may be due to the different living styles and dietary habits of the patients. Another possible explanation may be the difference in information, education and communication strategies; clinical as well as drug adherence level. In this study, significant association between sex and optimal BP control was observed. Accordingly, female patients were two times more likely to have optimal BP as compared to males. This result is in line with studies done in rural and urban communities in high, middle, and low income countries [9], Bangladesh [14], self-selected sub-Saharan African urban population [15], Nsukka, Nigeria [24], and Sudanese adults [23] where females were achieved optimal BP control than males. This may be due to males are usually loaded by activities outside the door which makes them tiring, exposes them to forget their drugs and finally make them difficult to control their blood pressure.

Age was another factor significantly associated with blood pressure control found in this study. Accordingly, hypertensive patients older than 60 years were three times more likely to control their blood pressure as compared to age group of 18–40 years. This finding is supported by study done in rural and urban communities in high, middle, and low income countries [9] and Macau, China [12]. This may be due to denial of the existence of the disease or becoming busy with activities outside the home in young patients that makes them forget to take medications.

Eating foods high in vegetables reduces blood pressure of hypertensive patients. The finding of this study showed association between eating vegetables on most days of the week and optimal BP control. Those hypertensive patients who ate vegetables on most days of the week were two times more likely to have optimal BP control as compared to patients who didn’t eat vegetables on most days of the week. The possible reason may be vegetables are good source of potassium and this result in decreasing blood pressure. Using top added salt on plate during meal preparation was significantly associated with blood pressure control in this study. Hence, patients who used top added salt on a plate were 80% less likely to have optimal BP control as compared to patients who didn’t use top added salt. This is similar with studies done in Macau, China [12] and Southern China [25]. This may be due to high salt intake causes fluid retention which increases cardiac burden resulting in high blood pressure.

Performing adequate physical activity have strong and independent role in reducing blood pressure. Consistent to what was previously reported from other sub-Saharan countries and southern China [15, 25], this study revealed patients who did adequate and low level of physical activity were more likely to have optimal BP control than patients with no physical activity. This may be due to regular physical activity being a significant factor in weight and blood pressure reduction.

Presence of asthma in patients with hypertension can worsen the conditions of the patient. This study revealed asthma was significantly and independently associated with blood pressure control. Asthmatic hypertensive patients were 67% less likely to have optimal BP control than non-asthmatic hypertensive patients which is supported by a study in KwaZulu-Natal, South Africa [26]. These may be due to side effects of some of the anti-asthmatic medications having a beta agonist effect on the heart (increasing heart rate and force of contraction) which results blood pressure to increase.

Number of drugs had a significant association with blood pressure control. This study revealed those patients who took less than three drugs a day were three times more likely to have optimal BP control as compared to patients who took three or more drugs a day. This study is in line with studies done in KwaZulu-Natal, South Africa [26], and Adama referral hospital in Ethiopia [16]. The possible reason may be patients who took two or fewer drugs may become more adhere to their drugs and finally their blood pressure becomes controlled. Adherence to prescribed medication was another factor significantly associated with blood pressure control in this study. Hypertension patients who were poorly adherent to their prescribed antihypertensive drugs were 82% less likely to have an optimal Bp control as compared to those who were adherent to their prescribed antihypertensive drugs. This study is in line with studies done in Malaysia [27], KwaZulu-Natal [26], and University of Gondar Hospital, Northwest Ethiopia [28]. The possible reason may be as the number of drugs they took increases; they may face a problem to take the right drug and dose at right time.

This study has the following limitations: Some key confounding variables such as physical and biochemical measurements were not included in the study. Self-reporting was used as the only method of measuring adherence and may have the disadvantages of recall bias. It is difficult to infer causation for some variables using a cross sectional study design.

## Conclusions

In conclusion, the proportion of hypertensive patients whose BP is optimally controlled was relatively low especially among male patients. Females, age group older than 60 years, vegetable consumption on most days of the week, adequate physical exercise, and taking less than three drugs per day were positively and significantly associated with optimal BP control whereas poor adherence to medications, having asthma comorbidity and use of top added salt on a plate were negatively and significantly associated with optimal BP control. Recognizing the fact that controlling BP reduces cardiovascular disease related morbidity and mortality, and prevents costly interventions, we recommend to policy makers in collaboration with stakeholders to develop strategies about the importance of lifestyle modifications, and adherence counseling to effectively control BP. Moreover, we suggest researchers to do further longitudinal studies that include physical and biochemical measurements to identify most important variables associated with blood pressure control and to look at the cause and effect relationship between variables and blood pressure control.

### Ethical approval and consent to participate

Ethical clearance was obtained from Ethical Review Board (ERB) of Institute of Public Health, College of Medicine and Health Science, University of Gondar. Permission letter was obtained from the hospital’s chief executive officer. Informed verbal consent was held from each participant to review their medical records and use the data and participant involvement in the study was on a voluntary basis. Confidentiality of data was kept by using identification numbers rather than names and limiting access to the data.

## Supporting information

S1 Quest This is the english version of the questionnaire. (DOCX)

S2 Quest This is the amharic version of the questionnaire. (DOCX)

S1 Data This is the data set of the study. (SAV)

## Abbreviations

ANRS: Amhara National Regional State
AOR: Adjusted Odd Ratio
BP: Blood Pressure
WHO: World Health Organization
