# Supplementary material for: Determinants of blood pressure control amongst hypertensive patients in Northwest Ethiopia
Source: PLoS One. 2018 May 2;13(5):e0196535. doi: 10.1371/journal.pone.0196535 (PMC5931630; doi:10.1371/journal.pone.0196535)
Supplement: S1 Quest — (DOCX) [file pone.0196535.s001.docx]

**English version of the Questionnaire**

**General information**

Serial no.______________ Card No______Kebele_____________

Data collection date________________________________

Name of data collector______________________________

Signature _______________________________________

**Section I: Assessment of Socio-demographic and economic variables**

| **S.No** | **Assessment of Socio-demographic and socio economic variables** | | |
| --- | --- | --- | --- |
|  | **Question** | **Response** | **Remark** |
| 101 | Sex | 1. Male 2. Female |  |
| 102 | How old are you? | ------------Years |  |
| 103 | What is your ethnic group? | 1. Amhara 2. Tigre 3. Agew 4. Others, specify----------------- |  |
| 104 | What is your religion? | 1. Orthodox 2. Muslim 3. Protestant 4. Others, specify----------------- |  |
| 105 | What is your marital status? | 1. Single 2. Married 3. Divorced 4. Widowed 5. Separated |  |
| 106 | What is the highest level of education you have completed? | 1. Unable to read and write 2. Read and write 3. Primary school 4. Secondary school 5. High school/preparatory school 6. College/University completed |  |
| 107 | Occupational status? | 1. Government employee 2. Retired 3. House wife 4. Daily laborer 5. Merchant 6. Farmer 7. No job 8. Others, specify ____________ |  |
| 108 | Residence? | 1. Urban 2. Rural |  |
| 109 | Average Family monthly income? | _________________ETB |  |

**Section II: Behavioural Measurements**

| **Tobacco Use** | | | | | |
| --- | --- | --- | --- | --- | --- |
| **S.No** | **Question** | | **Response** | **Remark** | |
| 201 | Do you ever smoke cigarettes? | | 1. Yes 2. No | If no skip to question 205 | |
| 202 | For how long do you smoke cigarettes? | | 1. Below 1 year 2. 1-5 year 3. >5 year |  | |
| 203 | Do you smoke daily? | | 1. Yes 2. No |  | |
| 204 | How many cigarettes per day? | | ---------Number |  | |
| 205 | Do you consume an alcoholic drink during the last 6 months? | | 1. Yes 2. No | | If not skip to Q209 |
| 206 | What type of alcohol did you drink? | | 1. Beer 2. Wine 3. Tella/Local beer 4. Areki 5. Others, specify____ | |  |
| 207 | On average how many glasses/bottles do you drink per day? | | _____bottles/glasses/birlie/Tassa | |  |
| 208 | How frequent did you drink alcoholic drink per week? | | ---------days | |  |
| 209 | | During the last 6 months, on most days of the week which type of diet did you eat? | 1. Meat 2. Fruits 3. Vegetables 4. Cereal products 5. Other, specify____ | |  |
| 210 | | What type of oil or fat is most often used for meal preparation in your household during the last 6 months? | 1. Vegetable oil 2. Butter 3. Sesame /nug oil 4. Others, specify__ | |  |
| 211 | | During the last 6 months do you used additional salt added on plate after food preparation? | 1. Yes 2. No | |  |
| 212 | | Does your work involve vigorous-intensity activity that causes large increases in breathing or heart rate like [carrying or lifting heavy loads, digging or construction work] during the last 6 months? | 1. Yes 2. No | | **If no skip to Q 215** |
| 213 | | In a typical week, on how many days do you do vigorous-intensity activities as part of your work? | _____days | |  |
| 214 | | How much time do you spend doing vigorous-intensity activities at work on a typical day? | _____Hours/minutes | |  |
| 215 | | Do you walk or use a bicycle (pedal cycle) to go to and from places in the last 6 months? | 1. Yes 2. No | | If No skip to Q 301 |
| 216 | | In a typical week, on how many days do you walk or bicycle to get to and from places? | _______ days | |  |
| 217 | | How much time do you spend walking or bicycling for travel on a typical day? | _____Hours/minutes | |  |

**Section III: Co-morbidities**

| **S.No** | **Question** | **Response** | **Remark** |
| --- | --- | --- | --- |
| 301 | Is there cardiovascular disease? | 1. Yes 2. No | From card records |
| 302 | Is there diabetes mellitus? | 1. Yes 2. No | " |
| 303 | Is there chronic kidney diseases? | 1. Yes 2. No | " |
| 304 | Is there asthma? | 1. Yes 2. No | " |
| 305 | Do you have family history of hypertension? | 1. Yes 2. No | By interviewing |
| 306 | Do you receive Health education/advice about your disease? | 1. Yes 2. No | " |

**Section IV: Medication related**

| **S.No** | **Question** | **Response** | | **Remark** |
| --- | --- | --- | --- | --- |
| 401 | What types of drug you take during the last 6 months? | 1. HCT 2. Enalapril 3. Nifidipine 4. Methyldopa 5. Propranolol 6. Atenolol 7. Others, specify | | From card review |
| 402 | How many drugs do you take per day? | ______ __drugs | |  |
| 403 | Medication adherence | Yes | No |  |
| 403 A | Do you ever forget to take your antihypertensive drugs? | 0 | 1 |  |
| 403B | Do you ever have problems remembering to take your antihypertensive medications? | 0 | 1 |  |
| 403C | When you feel better, do you sometimes stopped taking your antihypertensive medicine? | 0 | 1 |  |
| 403D | Sometimes if you felt worse when you take your antihypertensive medicine, do you stopped taking it? | 0 | 1 |  |

| **Section V: Blood pressure measurements** | | | |
| --- | --- | --- | --- |
| 501 | 6 months before the study | Systolic _____mmHg  Diastolic_____mmHg | From card review |
| 502 | 4 months before the study reading | Systolic _____mmHg  Diastolic_____mmHg |  |
| 503 | Recent reading | Systolic _____mmHg  Diastolic_____mmHg |  |
