# Supplementary material for: Determinants of blood pressure control amongst hypertensive patients in Northwest Ethiopia
Source: PLoS One. 2018 May 2;13(5):e0196535. doi: 10.1371/journal.pone.0196535 (PMC5931630; doi:10.1371/journal.pone.0196535)
Supplement: S2 Quest — (DOCX) [file pone.0196535.s002.docx]

**አጠቃላይ መረጃ**

**01. መለያ ቁጥር------------------------- ቀበሌ -----------------**

**02. መጠይቁ የተካሔደበት ቀን --------------------/--------------/----------------**

**03. የመረጃ ሰብሳቢው ስም----------------------------------------------- ፊርማ ---------------------------**

**ክፍል አንድ ማህበራዊና ስነ ህዝባዊ መረጃዎች**

| **ተ.ቁ** | **ጥያቄዎች** | **ምላሽ** | **ምርመራ** |
| --- | --- | --- | --- |
| 101 | ፃታ | 1. ወንድ 2. ሴት |  |
| 102 | እድሜዎ ስንት ነው? | -----------ዓመት |  |
| 103 | ብሔር? | 1. አማራ 2. ትግሬ 3. አገዉ   4. ሌላ ይገለጽ---------------- |  |
| 104 | ሐይማኖት? | 1. ኦርቶዶክስ 2. ሙስሊም 3. ፕሮቴስታንት 4. ሌላ ይገለጽ---------------- |  |
| 105 | የጋብቻ ሁኔታ? | 1. ያላገባ/ች 2. ያገባ/ች 3. የፈታ/ች 4. የሞተችበት/ባት 5. ተለያይተው የሚኖሩ |  |
| 106 | የትምህርት ደረጃ? | 1. ማንበብና መፃፍ የማይችል/የማትችል 2. ማንበብና መፃፍ የሚችል/የምትችል 3. የመጀመሪያ ት/ት (1-8) 4. 2ኛ ደረጃ ትምህርት (9-10) 5. መሰናዶ ትምህርት (11-12) 6. ኮሌጅ/ዩኒቨርሲቲ ያጠናቀቀ |  |
| 107 | የስራ ሁኔታ? | 1. የመንግስት ሰራተኛ 6. ገበሬ 2. ጡረተኛ 7. ስራ የሌለው 3. የቤት እመቤት 8. ሌላ ይገለጽ----- 4. የቀን ሰራተኛ 5. ነጋዴ |  |
| 108 | የመኖሪያ አድራሻ? | 1. ከተማ 2. ገጠር |  |
| 109 | አማካይ ወርሃዊ የቤተሰብ ገቢ? | --------------------የኢትዮዽያ ብር |  |

| **ክፍል ሁለት፡- ከስነ ባህሪ ጋር የተያየዙ ጥያቄዎች** | | | |
| --- | --- | --- | --- |
| ተ.ቁ | ጥያቄዎች | ምላሽ | **ምርመራ** |
| 201 | ሲጋራ አጭሰዉ ያዉቃሉ? | 1. አዎ 2. የለም | የለም ካሉ ወደ ጥያቄ 205 ይለፉ |
| 202 | ለምን ያክል ጊዜ አጭሰዉ ያዉቃሉ? | 1. ከ 1 አመት በታች 2. ከ 1-5 አመት 3. ከ 5 አመት በላይ |  |
| 203 | በየቀኑ ያጨሻሉ? | 1. አዎ 2. የለም |  |
| 204 | በቀን ምን ያክል ሲጋራ ያጨሻሉ? | ---------በቁጥር |  |
| 205 | በዚህ 6 ወር ዉስጥ የአልኮል መጠጦችን ጠጥተዉ ያዉቃሉ? | 1. አዎን 2. የለም | መልሱ የለም ከሆነ ወደ ጥያቄ 209 ይለፉ |
| 206 | ምን አይነት የአልኮል መጠጦችን ተጠቅመዉ ያዉቃሉ? | 1. ቢራ 3. ጠላ 2. ወይን 4. አረቂ   5.ሌላ ይገለጽ------- |  |
| 207 | በቀን ዉስጥ ምን ያክል የአልኮል መጠጥ ተጠቅመዉ ያዉቃሉ? | ----------ጠርሙስ/ብርጭቆ/ብርሌ/ጣሳ  ባለ---------ሚሊ ሊትር |  |
| 208 | በሳምንት ዉስጥ ስንት ቀን ይጠጣሉ? | ----- ቀን |  |
| 209 | አዘዉትረዉ የሚመገቡት ምግብ ምንድን ነዉ? | 1. ስጋ 2. ፍራፍሬ 3. ቅጠላቅጠል 4. የእህል ዉጤቶች 5. ሌላ ይገለጽ------------ | ከ 1 በላይ መመለስ ይቻላል |
| 210 | አብዛኛውን ጊዜ ለምግብ ዝግጅት ምን አይነት ዘይት ወይም ቅባት ተጠቅመዉ ያዉቃሉ? | 1. የሚረጋ ዘይት 2. ቅቤ 3. የሲሊጥ /የኑግ ዘይት 4. ሌላ ይገለጽ------------- |  |
| 211 | በዚህ 6 ወር ዉስጥ ጨዉ ያለበት ምግብ ተጠቅመዉ ያዉቃሉ? | 1. አወ 2. የለም |  |
| 212 | በዚህ 6 ወር ዉስጥ የሚሰሩት ስራ ብዙ ጉልበትና ሀይል የሚጠይቅ የልብ ምትንና የአተነፋፈስን ፍጥነት የሚጨምር ነውን? ማለትም መሸከም፣ መቆፈር፣ወይም ግንባታን ወዘተ | 1. አዎ 2. የለም | መልሱ የለም ከሆነ ወደ ጥያቄ 215 ይለፉ |
| 213 | በሳምንት ውስጥ ምን ያህል ቀናት ጉልበትና ኃይል የሚጠይቅ ስራ ይሰራሉ? | _____ቀናት |  |
| 214 | በቀን ውስጥ ምን ያህል ደቂቃ/ሰዓት ከባድ ጉልበትና ሃይል የሚጠይቅ ስራ እየሰሩ ያሳልፋሉ? | ------- በሰዓት/በደቂቃ |  |
| 215 | ባለፈዉ 6 ወር ዉሰጥ ወደ ተለያዩ ስፍራዎች ለመድረስ/ለመንቀሳቀስ በእግር ጉዞ አድርገዉ ያዉቃሉ? | 1. አዎ 2. የለም |  |
| 216 | በሳምንት ውስጥ ምን ያህል ቀናት ወደ ተለያዩ ስፍራዎች ለመድረስ ወይንም ለመንቀሳቀስ በእግር ጉዞ አድርገዉ ያዉቃሉ? | --------ቀናት |  |
| 217 | በቀን ውስጥ ምን ያህል ሰዓት/ደቂቃ በእርምጃ ከቦታ ቦታ በመዘዋወር የሳልፋሉ | ----በሰዓት/በደቂቃ |  |

| **ክፍል አራት፡- መድሃኒትን በተመለከተ** | | | ምላሽ | |  |
| --- | --- | --- | --- | --- | --- |
| 401 | | በዚህ 6 ወር ዉሰጥ የወስዱት የደም ገፊት መድሃኒት አይነት | 1. HCT 2. Enalapril 3. Nifidipine 4. Methyldopa 5. Propranolol 6. Atenelol 7. ሌላ ይገለጽ------- | | ከካርድ በማት (from the card) |
| 402 | ላለፉት 6 ወራት በቀን በቀን ምህ ያክል መድሃኒት ወስደዉ ያዉቃሉ? | | ------- -----በቁጥር | |  |
| 403 | የደም ገፊት መድሀኒትን በአግባቡ እና በተገቢዉ ሰአት መዉሰድን በተመለከተ | | አዎ | የለም |  |
| 403ሀ | የደም ገፊት መድሀኒትዎን ረስተዉ ያዉቃሉ? | | 0 | 1 |  |
| 403ለ | የደም ገፊት መድሀኒትዎን እንዳይወስዱ የከለከለዎት የሚያስታዉሱት የገጠመዎት ችግር አለ? | | 0 | 1 |  |
| 403h | አንዳንዴ ሻል ባለዎት ጊዜ የደም ግፊት መድሀኒትዎን አቁመዉት ያዉቃሉ? | | 0 | 1 |  |
| 403m | አንዳንዴ ህመሙ ባስ ባለብዎት ጊዜ መድሀኒቱን አቁመዉት ያዉቃሉ? | | 0 | 1 |  |

| **ክፍል 5: Blood pressure measurements** | | | |
| --- | --- | --- | --- |
| 501 | 6 months before the study | Systolic _____________ mmHg  Diastolic_____________ mmHg | ከካርድ በማየት |
| 502 | 4 months before the study reading | Systolic ______________mmHg  Diastolic______________mmHg |  |
| 503 | recent reading | Systolic _____________ mmHg  Diastolic______________mmHg |  |

| **ክፍል፡-ሶስት ተዛማጅ በሽታዎችን በተመለከተ** | | | |
| --- | --- | --- | --- |
| **ተ.ቁ** | **ጥያቄ** | **መልስ** | **ምርመራ** |
| 301 | የልብ ድካም በሽታ | 1. አለ 2. የለም | ከካርድ የሚወሰድ |
| 302 | የስኳር በሽታ | 1. አለ 2. የለም | ከካርድ የሚወሰድ |
| 303 | የቆየ የኩላላት በሽታ | 1. አለ 2. የለም | ከካርድ የሚወሰድ |
| 304 | የአስም በሽታ | 1. አለ 2. የለም | ከካርድ የሚወሰድ |
| 305 | በወላጆችዎመካከል የደም ግፈት መጨመር ያለበት ሰው አለ? | 1. አዎ 2. የለም | በመጠየቅ |
| 306 | ስለበሽታዎ የጤና ትምህርት ተነግሮዎት ያውቃል? | 1. አዎ 2. የለም | በመጠየቅ |
